# Supplementary material for: The genome sequence of the European smelt, Osmerus eperlanus (Linnaeus, 1758)
Source: Wellcome Open Res. 2025 Mar 4;10:118. [Version 1] doi: 10.12688/wellcomeopenres.23789.1 (PMC11966098; doi:10.12688/wellcomeopenres.23789.1)
Supplement: Peer review history

# Reviewer report: Reviewer response for version 1

**DOI:** 10.21956/wellcomeopenres.26238.r120820

**Published:** 2025-04-05

**Authors:** Le Wang

**Affiliations:** 1 Temasek Lifesciences Laboratory, Singapore, Singapore

This paper presents the sequencing and assembly of a chromosome-level genome of the European smelt, a fish species of ecological and economic importance. The manuscript is well-prepared, with a clear and coherent presentation of both methods and results. However, I recommend that the authors provide more comprehensive genome annotation, particularly with regard to repetitive elements and protein-coding genes. Overall, I believe this work is valuable to the scientific community. Aside from this suggestion, I have no major concerns or questions about the manuscript.

Are sufficient details of methods and materials provided to allow replication by others?

Yes

Is the rationale for creating the dataset(s) clearly described?

Yes

Are the datasets clearly presented in a useable and accessible format?

Yes

Are the protocols appropriate and is the work technically sound?

Yes

Reviewer Expertise:

Fish genetic and genomics, evolutionary biology, aquaculture genetics

I confirm that I have read this submission and believe that I have an appropriate level of expertise to confirm that it is of an acceptable scientific standard.

# Reviewer report: Reviewer response for version 1

**DOI:** 10.21956/wellcomeopenres.26238.r120397

**Published:** 2025-04-02

**Authors:** Yniv Palti

**Affiliations:** 1 USDA-ARS National Center for Cool and Cold Water Aquaculture, Kearneysville, WV, USA

The report provides brief and sufficient rationale for the genome assembly of the European smelt as part of the Darwin Tree of Life project. This anadromous fish species is commercially important with some local populations threatened by polluted habitat. The tree of Life project aims to sequence all named eukaryotic species in the Atlantic Archipelago of Britain and Ireland.

 Over all the methods are described clearly and succinctly. I gave it a partly mark only because the citations in support of two of processes reported for the assembly curation are articles in preparation.

 The information reported in Table 2 and Figure 2 is redundant with some very minor discrepancies such as the BUSCO (94.9% in the table and 95.2% in the Figure).

 Other than that all methods an protocols are sound and adequate references are provided for additional technical information. The assembly chromosome sequences and support raw data are publicly available for downloading from EBI. The quality of this technical report is definitely sufficient for indexing.

Are sufficient details of methods and materials provided to allow replication by others?

Partly

Is the rationale for creating the dataset(s) clearly described?

Yes

Are the datasets clearly presented in a useable and accessible format?

Yes

Are the protocols appropriate and is the work technically sound?

Yes

Reviewer Expertise:

Genomics resources and tools and genome assemblies. Fish genetics and aquaculture breeding.

I confirm that I have read this submission and believe that I have an appropriate level of expertise to confirm that it is of an acceptable scientific standard.

# Reviewer report: Reviewer response for version 1

**DOI:** 10.21956/wellcomeopenres.26238.r121374

**Published:** 2025-03-31

**Authors:** Steven M Mussmann

**Affiliations:** 1 Southwestern Native Aquatic Resources and Recovery Center, Dexter, USA

Fletcher *et al*. report on the assembly of a reference genome for European Smelt, *Osmerus eperlanus*. They provide a brief background on the life history, distribution, and commercial importance of the species. They also describe the methods for assembling what appears to be a chromosome-level genome assembly for the species from long-read PacBio HiFi data and short-read Hi-C data. 

 The authors state that genome sequencing of this organism was done as part of a larger Darwin Tree of Life Project to sequence eukaryotic species from Britain and Ireland. If there are additional advantages for having a reference genome for this species it would be great if the authors could include those as well. 

 All work seems to follow modern and scientifically sound methods for collecting tissues, conducting lab work, and conducting *de novo* assembly of a reference genome. 

 I answered ‘yes’ to the question “Are sufficient details of methods and materials provided to allow replication by others?”. I think someone could replicate the work done here from their descriptions. However, I also think it would be advantageous if the authors are able to post any scripts written to conduct the genome assembly procedures on a public site such as Github. 

 The authors have made all raw data and the assembly available in public databases. 

**Minor comments:**

- In the sentence beginning “The specimen was collected by Chris Fletcher (Natural History Museum…” the authors need a close parenthesis after “Museum.”
- In the sentence “DNA concentration was quantified using the Qubit Fluorometer v2.0 and Qubit HS Assay Kit and the final library fragment size analysis was carried out using the Agilent Femto Pulse Automated Pulsed Field CE Instrument and gDNA 165kb gDNA and 55kb BAC analysis kit,” is ‘gDNA’ supposed to be repeated toward the end of the sentence? (i.e., …and gDNA 165kb gDNA and…)

Are sufficient details of methods and materials provided to allow replication by others?

Yes

Is the rationale for creating the dataset(s) clearly described?

Yes

Are the datasets clearly presented in a useable and accessible format?

Yes

Are the protocols appropriate and is the work technically sound?

Yes

Reviewer Expertise:

Population genetics and genomics; Bioinformatics

I confirm that I have read this submission and believe that I have an appropriate level of expertise to confirm that it is of an acceptable scientific standard.
